# Sustainability of Positive Body Image Changes One Year After Exercise Interventions in Young Women: A Quasi-Experimental Study

**DOI:** 10.3390/bs16010083

**Published:** 2026-01-07

**Authors:** Rasa Jankauskiene, Vaiva Balciuniene, Renata Rutkauskaite, Migle Baceviciene

**Affiliations:** 1 Institute of Sport Science and Innovations, Lithuanian Sports University, 44221 Kaunas, Lithuania; rasa.jankauskiene@lsu.lt; 2 Department of Physical and Social Education, Lithuanian Sports University, 44221 Kaunas, Lithuania; vaiva.balciuniene@lsu.lt (V.B.); renata.rutkauskaite@lsu.lt (R.R.)

**Keywords:** positive body image, intervention, embodiment, physical activity, body functionality, mindfulness, follow-up

## Abstract

Although some exercise interventions have demonstrated short-term benefits for women’s positive body image, evidence regarding their longer-term effects—particularly under real-world conditions—remains limited. Understanding the sustainability of post-intervention outcomes is important for assessing the practical relevance of exercise programmes and their potential to improve positive body image. The aim of this study was to examine the sustainability of post-intervention outcomes related to positive body image one year after participation in non-randomised 8-week Nirvana Fitness (NF) and Functional Training (FT) interventions among young women under real-world conditions. Young women (mean age 22.79 ± 6.14) were self-selected into either the NF group (n = 16) or the FT (n = 15) group and participated in an eight-week exercise intervention. A control group (n = 17) of women did not participate in the intervention. Participants completed online questionnaires assessing body appreciation, body surveillance, functionality appreciation, body–mind connection, intrinsic exercise motivation, physical activity at baseline, immediately after the intervention, and 12 months later. Changes in outcomes over time were analysed using linear mixed-effects models with fixed effects for group, time, and their interaction, random intercepts for participants, and adjustment for age and body mass index. Analysis revealed significant group × time interactions for body appreciation, functionality appreciation, body–mind connection, and intrinsic exercise regulation, indicating differential changes over time between interventions and control groups. Body surveillance showed a significant effect of time only, whereas leisure-time exercise differed between groups but did not change over time. Overall, intervention groups demonstrated more favourable change patterns across positive body image-related outcomes compared with the control group. Conclusion: Participation in intervention programmes was associated with sustained improvements in positive body image and exercise motivation, but not with changes in body surveillance or leisure-time physical activity. Given the self-selected group allocation and small sample size, these findings should be considered exploratory. Larger randomised studies are needed to confirm the sustainability and generalisability of these findings.

## 1. Introduction

### 1.1. Importance of Positive Body Image for Women’s Physical Activity 

For a considerable number of women, body image constitutes a significant component of quality of life and mental well-being (Baceviciene et al., 2020; Frederick et al., 2022; Linardon et al., 2022; Swami et al., 2023). It is a multidimensional construct encompassing perceptual, cognitive, affective, and behavioural components that form a mental representation of an individual’s physical appearance and body functionality (Cash & Smolak, 2011; Vani et al., 2021). A substantial proportion of extant research on women’s body image has historically concentrated on body dissatisfaction and the sociocultural forces that promote adherence to idealised appearance standards. In contrast, more recent scholarship conceptualises positive body image as a separate and comprehensive construct, characterised by women’s capacity to accept, value, and care for their bodies while actively resisting appearance-based sociocultural pressures (Tylka & Wood-Barcalow, 2015b). A mounting body of evidence indicates that positive body image is associated with enhanced self-esteem and psychological well-being, along with a reduced likelihood of experiencing body dissatisfaction and eating disorders (Linardon, 2021; Linardon et al., 2022). Among women, higher positive body image is associated with more intuitive approaches to eating, higher engagement in body functionality and health related goals for health-promoting physical activity and greater physical activity (Avalos & Tylka, 2006; Homan & Tylka, 2014; Linardon et al., 2021; Tylka & Homan, 2015). 

Negative body image can serve as a significant barrier to women’s participation in physical activity, as body dissatisfaction and body surveillance—the habitual monitoring of one’s physical appearance from an observer’s perspective—may diminish enjoyment and disrupt the experience of flow during exercise (Baceviciene et al., 2024; Cowley & Schneider, 2025; Embacher-Martin & McGloin, 2025; Engeln et al., 2023; More et al., 2019). When women habitually monitor their appearance and observe their bodies from a third-person perspective, they tend to become less attuned to internal bodily cues. They also become less present in the activity itself and make less favourable judgements about how they will feel when participating in future physical activity or recalling past experiences associated with it (Engeln et al., 2023; Fredrickson & Harrison, 2005; Gilchrist et al., 2021). This externally oriented focus interferes with the ability to experience intrinsic enjoyment during exercise and may, over time, decrease persistence in regular physical activity (Pila et al., 2021). 

Conversely, cultivating positive body image helps shift attention from how the body looks to how it feels and functions (Tylka & Wood-Barcalow, 2015b). Positive body image is related to more autonomous forms of motivation and greater enjoyment that are background for exercise persistence (Cox et al., 2019; Homan & Tylka, 2014; Hurst et al., 2017; Ryan et al., 2009; Tylka & Homan, 2015; Vani et al., 2021). Thus, promoting positive body image is not only beneficial for women’s psychological well-being but also for sustained engagement in physical activity. Movement-based interventions that emphasise bodily awareness, functionality, and mindful engagement—rather than appearance-based outcomes—may be particularly effective in supporting these processes (Matheson et al., 2023). One prominent example of such approaches is mindfulness-based physical activity, which has been widely studied in relation to women’s body image (Ullrich-French et al., 2022).

Mindfulness-based physical activities such as yoga have consistently demonstrated beneficial effects on women’s body image and psychological well-being (Alleva et al., 2020; Ariel-Donges et al., 2019; Borden & Cook-Cottone, 2020; Cook-Cottone et al., 2020; Cox & McMahon, 2019; Halliwell et al., 2019). These practices combine movement, conscious breathing, and mental focus, facilitating non-judgemental awareness of body sensations and reducing body surveillance (Cox et al., 2016, 2017). The Developmental Theory of Embodiment (DTE) (Piran, 2019) offers an understanding of the relationship between women’s lived experiences of their bodies and their overall well-being and body image. According to the theory, positive embodiment occurs when women experience comfort, connection and agency in their bodies; feel free from self-objectification (including body surveillance); and are able to engage in joyful, empowering movement. From this perspective, physical activities that emphasise internal awareness, competence and appreciation of body functionality can foster a stronger sense of embodiment and positive body image (Menzel et al., 2019; Piran & Neumark-Sztainer, 2021). It is important to note that mindfulness-based processes offer a complementary explanation for how such internally focused movement experiences support embodiment. The Monitoring and Acceptance Theory (MAT) (Lindsay & Creswell, 2017) provides a further explanation of the mechanisms through which mindfulness improves psychological well-being. According to MAT, the operational mechanism of mindfulness involves two complementary processes: monitoring, which entails the sustained attention to present-moment internal experiences, and acceptance, which involves the adoption of a non-judgemental and open attitude towards those experiences. When applied to movement, these processes facilitate women in observing their body sensations without evaluating them, maintaining mental presence and ceasing self-criticism or preoccupation with appearance (Ullrich-French et al., 2022). The focus on acceptance and introspection cultivates a heightened appreciation for the body, thereby reducing self-objectification. This, in turn, fosters the development of a more connected and compassionate relationship with the body. 

Observational and intervention studies concluded that while participation in yoga directly affects body appreciation, it may increase mindfulness (Cox et al., 2016; Cox & McMahon, 2019; Gaiswinkler & Unterrainer, 2016; Hewett et al., 2011), positive embodiment and body and mind connectedness (Alleva et al., 2020; Halliwell et al., 2019), reduce self-objectification (Alleva et al., 2020; Cox et al., 2016; Mahlo & Tiggemann, 2016), increase physical fitness and self-concept (Cox et al., 2016; Hewett et al., 2011) and internal reasons for exercise (Cox et al., 2016). All these variables are related to increased positive body image (Linardon et al., 2022; Swami et al., 2023; Tylka & Wood-Barcalow, 2015b) and positive embodiment (Piran & Neumark-Sztainer, 2021). However, the majority of extant research originates from yoga-based interventions, and research on other mindfulness-oriented exercise modalities remains limited. 

### 1.2. Effects of Mindfulness-Based Physical Activity on Women’s Positive Body Image

While yoga and similar mindfulness-based practices have been extensively studied for their benefits to women’s body image (Alleva et al., 2020; Ariel-Donges et al., 2019; Borden & Cook-Cottone, 2020; Cook-Cottone et al., 2020; Cox & McMahon, 2019; Halliwell et al., 2019), other contemporary exercise modalities may also cultivate embodiment and foster positive body image (Menzel & Levine, 2011). Examples of these modalities are Nirvana Fitness (NF) and Functional Training (FT). FT is a modern, group-based fitness modality that emphasises multi-joint, full-body movements replicating daily physical activities and is designed to improve strength, balance, coordination, proprioception, and overall functional performance (Beckham & Harper, 2010). Conducted at moderate to vigorous intensity, FT incorporates progressive, weight-bearing, and multiplanar exercises that enhance both physical and neuromuscular efficiency (Xiao et al., 2021). FT has been shown to improve cardiovascular parameters in adults (Rezende Barbosa et al., 2018), and to positively influence speed, muscular strength, power, balance, agility, and overall performance in athletes (Wang et al., 2023; Xiao et al., 2021) as well as in university students (W. J. Zhang et al., 2025). In women, high-intensity FT has been associated with improvements in muscular endurance, upper-body strength, lower-body power, and agility, alongside increases in body mass and reductions in body fat (Sobrero et al., 2017) These findings suggest that FT may positively affect body image by promoting measurable improvements in physical fitness and body composition. 

Further, focusing on body functionality and performance rather than appearance during exercise may encourage women to direct their attention toward bodily capabilities, a process that has been theoretically and empirically linked to more positive body image (Alleva et al., 2020; Linardon et al., 2023; Menzel & Levine, 2011; Tylka & Wood-Barcalow, 2015b). According to Self-Objectification Theory (Fredrickson & Harrison, 2005; Fredrickson & Roberts, 1997), monitoring one’s appearance from an observer’s perspective undermine physical performance (Fredrickson & Harrison, 2005) and disrupt mood, body satisfaction, and body appreciation during exercise (Baceviciene et al., 2024; Engeln et al., 2023). In contrast, a stronger body–mind connection is associated with lower levels of self-objectification and body surveillance (Menzel & Levine, 2011). Also, body functionality-focused athletic activity might increase positive embodying experiences through increased interoceptive awareness (Menzel & Levine, 2011; Todd et al., 2019; Wallman-Jones et al., 2021) which is related to increased positive body image (Wallman-Jones et al., 2021). These findings suggest that engagement in physical activity may foster a more internal body orientation, reduce the tendency to view the body from a third-person perspective, and promote more positive body image experiences during exercise.

In contemporary wellness contexts, a range of yoga-based and mindful movement practices have emerged. These span traditional approaches such as yoga, tai chi and qigong, as well as more recent practices including Pilates. These methodologies generally encompass low- to moderate-intensity physical exertion and place emphasis on bodily awareness, proprioceptive attention, and a non-judgemental mental focus (La Forge, 2005). Studies showed that mind and body movement interventions increase sleep quality, reduce anxiety and stress, enhance emotional regulation, and overall wellbeing (Neuendorf et al., 2015; G. Zhang et al., 2025). Converging evidence from neuroimaging research further suggests that mind–body exercise may support neural processes underlying emotion regulation, interoception, and self-related processing, providing a plausible neurobiological mechanism through which such practices may promote adaptive body-related experiences and psychological well-being (Han et al., 2023). 

NF is a structured, yoga and Pilates based movement programme incorporating rhythmic movement sequences and guided breathing techniques. The programme’s stated aim is the promotion of bodily awareness and relaxation. NF places particular emphasis on regulated breathing patterns, including slower diaphragmatic breathing and prolonged exhalation. These breathing patterns have been associated with increased parasympathetic activation and improved interoceptive awareness in mindfulness- and breath-focused practices more broadly (Brenner et al., 2020; Khng, 2023; Paulus, 2013). Such breathing and attentional components are consistent with mechanisms identified in mindfulness-based movement interventions, which have been associated with increases in body appreciation, body–mind connectedness, and positive mood (Halliwell et al., 2019) as well as reductions in self-objectification after participation in yoga interventions (Alleva et al., 2020; Ariel-Donges et al., 2019; Cox et al., 2016; Halliwell et al., 2019). Accordingly, in the present study, NF is conceptualised as a mindfulness-oriented movement intervention rather than as a fitness programme per se, reflecting its emphasis on bodily awareness and attentional processes rather than physical conditioning outcomes. 

In our previous quasi-experimental study, we examined the impact of an eight-week positive body image programme on young women (Jankauskiene et al., 2024). We found significant improvements in body appreciation, functionality appreciation, and the connection between body and mind, as well as a decrease in body surveillance in the FT group. Improvements in the connection between body and mind, as well as body surveillance, were observed in the NF group, while no improvements were observed in the CN group. The effect of FT on body appreciation appeared to operate via improved mind–body connection in the FT and NF groups, and decreased body surveillance in the FT group (Jankauskiene et al., 2024). 

Despite their popularity, both NF and FT remain under-explored in scientific research examining body image outcomes, particularly from a long-term perspective. While there is growing evidence supporting the short-term benefits of mindfulness-based exercise interventions in improving women’s positive body image, most studies lack follow-up periods. As a result, it remains unclear whether observed improvements in positive body image and related constructs are maintained once structured interventions end, particularly under real-world conditions. Moreover, comparatively little research has examined the long-term sustainability of outcomes associated with positive body image. Addressing this gap is important for understanding whether such interventions have practical relevance for supporting sustained positive body image and continued engagement in physical activity among young women (Matheson et al., 2023; Vani et al., 2021). 

### 1.3. The Current Study

In our previous research we examined the immediate effects of an eight-week NF and FT intervention on young women’s positive body image and its correlates (Jankauskiene et al., 2024). The present study aimed to examine the sustainability of post-intervention outcomes under real-world conditions of the same eight-week non-randomised NF and FT interventions on young women’s positive body image (operating as body appreciation), functionality appreciation, body and mind connection, body surveillance, intrinsic motivation, and leisure-time physical activity. Specifically, we examined whether the improvements identified at post—test (Time 2) were maintained at a 12-month follow-up (Time 3). We hypothesised that enhancements in positive body image and its correlates following participation in NF and FT would be maintained to at least some extent 12 months after the intervention. Specifically, we expected that the following outcomes would be sustained at the 12-month follow-up: increased body appreciation, body functionality appreciation, body and mind connection, intrinsic exercise motivation and a decrease in body surveillance. No specific assumptions were formulated regarding physical activity at follow-up. 

## 2. Materials and Methods

### 2.1. Procedure and Participants

We conducted a non-randomised controlled trial with a longitudinal design involving three groups and three measurement points: baseline (pre-test), immediately after the 8-week intervention (post-test), and 12 months after the intervention (follow-up). Given the applied, real-world nature of the intervention and participants’ preferences for specific exercise modalities, a non-randomised quasi-experimental design was employed, consistent with methodological recommendations for behavioural and community-based interventions (Rosenbaum, 2020). The permission to implement the study was issued by the Ethics Committee of the Lithuanian Sports University (Protocol No. SMTEK-131). All participants provided written informed consent prior to participation. 

The study procedure has been described in detail elsewhere (Jankauskiene et al., 2024). Briefly, participants were allocated to one of three groups: Nirvana Fitness (NF), Functional Training (FT), or a non-intervention control group (CN). Allocation to the intervention groups was non-randomised; participants self-selected into NF or FT based on availability and preference. This approach was necessary due to practical constraints related to the student sample and fixed scheduling of physical activity sessions at a private sport club. Random allocation would have conflicted with academic commitments and was expected to substantially reduce recruitment and retention. Allowing self-selection enabled feasible implementation and maximised participation under real-world conditions, which aligns with recommendations for complex behavioural interventions (Craig et al., 2008). Participants were instructed to refrain from initiating new structured exercise programmes during the intervention period.

The NF and FT participants attended supervised group sessions twice weekly for eight consecutive weeks. Identical online self-report questionnaires were administered at baseline (pre-test, Time 1), immediately after the intervention (post-test, Time 2), and 12 months after the intervention (follow-up, Time 3) to evaluate the persistence of changes in positive body image and its correlates. The follow-up assessment was conducted by sending a link to the questionnaire online. For the present longitudinal analysis, participants who completed all three measurement points were included in the final dataset. There were no significant differences in all out-come measures assessed at the 8-week post-intervention time point between participants who completed the 12-month follow-up and those who were lost to follow-up.

**Figure 1:**
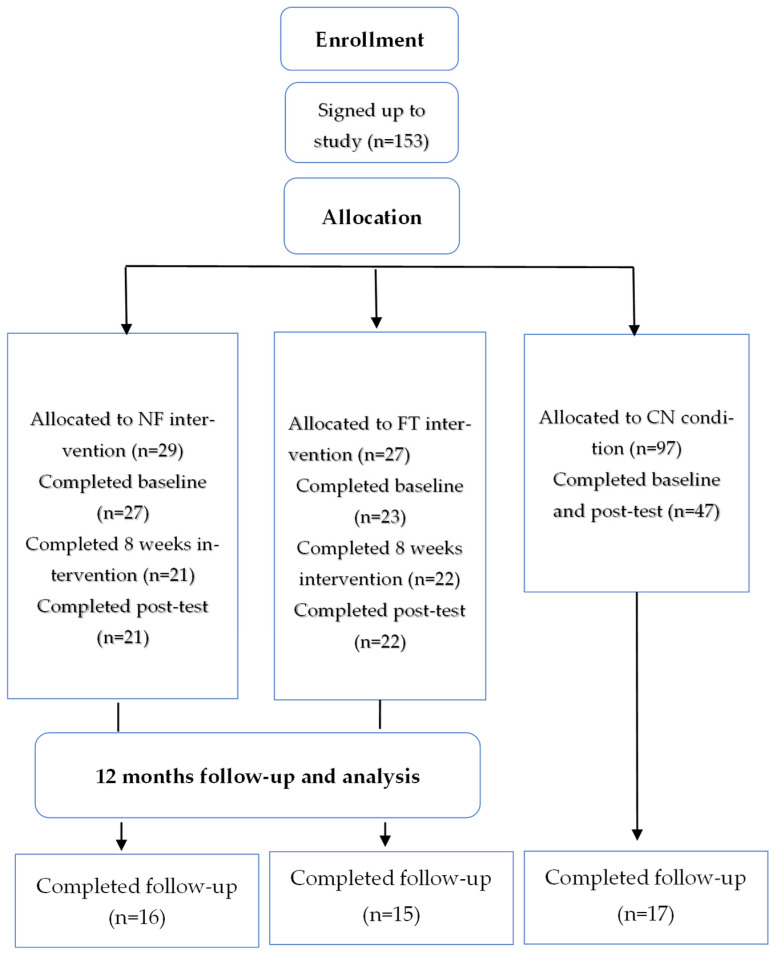
Participants flow throughout the study. Note. NF—nirvana fitness group, FT—Functional Training group, CN—control group.

In total, 48 women completed all three assessments and were included in the final analyses: 16 young women participated in the NF group, 15 in the FT group, and 17 in the CN group (Figure 1). Nearly half of the participants were living with a partner or married (47.9%), while 35.4% were single. Most participants were employed, with 47.9% working full-time and 31.3% part-time, whereas 20.8% were not employed. At the 12-month follow-up, there were no significant between-group differences in age or body mass index. Mean age was 24.60 ± 3.14 years in the functional training group, 25.94 ± 7.78 years in the Nirvana Fitness group, and 22.88 ± 3.12 years in the control group (*p* = 0.245). Mean body mass index was 22.81 ± 4.12 kg/m^2^, 22.63 ± 3.71 kg/m^2^, and 22.95 ± 4.87 kg/m^2^ in the functional training, Nirvana Fitness, and control groups, respectively, with no significant between-group differences (*p* = 0.977).

### 2.2. Intervention and Follow Up

The 8-week intervention consisted of two 60 min supervised sessions per week (total = 16 h), led by qualified instructors with experience in health and fitness training. Instructors were asked to refrain from appearance-based motivational language. Participants in the NF programme emphasised body–mind connection, diaphragmatic breathing, and relaxation through yoga- and Pilates-based movements performed in rhythmic flow with music; intensity of physical activity was low. The FT program targeted overall functionality and strength through multi-joint, full-body exercises at moderate intensity, incorporating weekly progressions such as stability and mobility drills, core training, resistance circuits, and TRX-based functional movements. The content of the interventions was standardised (Jankauskiene et al., 2024). Intervention fidelity was monitored using instructor-completed session checklists. Participant attendance was recorded in person at the sports club prior to each session and subsequently logged online. Standardised weekly email reminders were sent to support engagement, and participants who missed two consecutive sessions without prior notice received a personal SMS reminder. in the CN group did not take part in any structured exercise intervention during the study period. 

Subsequent to the conclusion of the 8-week intervention, no supplementary intervention content, guidance, or restrictions were imparted during the 12-month follow-up period. It was recommended to the participants that they should resume their usual daily routines. Engagement in other exercise programmes or body image–related interventions was not monitored during this period.

### 2.3. Study Measures

The demographic characteristics of participants included their age, the type of higher education institution they attended (university or college), their level of study, their marital status and their employment status.

Body appreciation was measured using the Lithuanian version of the Body Appreciation Scale-2 (BAS-2; Tylka & Wood-Barcalow, 2015a). This scale measures a person’s acceptance of, respect for, and favourable opinions of their own body, as well as their resistance to stereotyped appearance pressures. The BAS-2 comprises ten statements that are rated on a five-point scale ranging from one (never) to five (always). Examples of items are: ‘My body has at least some good qualities’; ‘I am attentive to my body’s needs’. An overall score was calculated by averaging all item responses, with higher averages reflecting stronger body appreciation. Previous research has shown that the Lithuanian adaptation has good psychometric quality (Baceviciene & Jankauskiene, 2020). In the present study, internal consistency at the pre-test, post-test and follow-up was excellent (Cronbach’s α = 0.96; 0.96 and 0.95, respectively).

Body functionality appreciation was measured using the Lithuanian version of the Functionality Appreciation Scale (FAS; Alleva et al., 2017). The scale measures an individual’s appreciation for what their body can do and what it is capable of (e.g., “I am grateful for the health of my body, even if it isn’t always as healthy as I would like it to be”, “I feel that my body does so much for me”). It comprises seven items, which are rated on a 5-point scale ranging from 1 (strongly disagree) to 5 (strongly agree), with higher scores representing greater appreciation of body functionality. The Lithuanian version has demonstrated adequate psychometric properties (Baceviciene et al., 2025). At the pre-test, post-test and follow-up internal consistency was excellent (Cronbach’s α = 0.92; 0.92 and 0.93, respectively).

The connection between the body and mind was assessed using the five-item Mind–Body Connection subscale of the Physical Activity Body Experiences Questionnaire (PABEQ; Menzel et al., 2019). This subscale contains five items reflecting the interaction between thoughts, energy, physicality, awareness, and the sense of self (e.g., I have a deep connection with my body, one that makes me feel powerful and effective). Items are rated on a 7-point Likert scale ranging from 1 (not at all true about me) to 7 (very true about me) with higher values reflecting stronger embodiment. The subscale demonstrated excellent internal consistency at pre-test, post-test, and follow-up (Cronbach’s α = 0.93, 0.89, and 0.91, respectively).

Body surveillance was measured using the Lithuanian version of the eight-item Body Surveillance (BS) subscale from the Objectified Body Consciousness Scale, OBC (McKinley & Hyde, 1996). Items were rated on a 7-point Likert scale ranging from 1 (strongly disagree) to 7 (strongly agree). Item examples are: “I think it is more important that my clothes are comfortable than whether they look good on me; During the day, I think about how I look many times”. Six items out of eight are reverse-scored. Higher scores indicate higher body surveillance. The Lithuanian adaptation of the BS has shown satisfactory psychometric properties among young women (Urvelyte, 2023). The internal consistency at pre-test was good (Cronbach’s α = 0.82).

Leisure-time physical activity was measured using the Godin and Shepard Leisure-Time Exercise Questionnaire (LTEQ), a self-report tool that estimates weekly exercise engagement (Godin, 2011). Participants indicated how often they performed strenuous, moderate or mild activities for at least 15 min at a time during a typical week. Examples of strenuous activities include running and hockey; moderate activities include baseball, tennis, easy cycling, volleyball, badminton and easy swimming; and mild activities include light walking and yoga. Each category was assigned a metabolic equivalent weighting (strenuous = 9, moderate = 5, mild = 3), and the weighted frequencies were summed to produce a total leisure-time activity score. Higher scores represent greater levels of self-reported physical activity.

Intrinsic exercise motivation was measured using the four-item Intrinsic motivation subscale of the Lithuanian version of the Behavioural Regulation in Exercise Questionnaire-2, BRERQ-2 (Markland & Tobin, 2004). Four items of Intrinsic motivation subscale are: “I exercise because it’s fun; I enjoy my exercise sessions, I find exercise a pleasurable activity and I get pleasure and satisfaction from participation in exercise”. Participants rated each statement on a 5-point Likert scale, ranging from 1 (not true for me) to 5 (very true for me). Responses were averaged to create an overall intrinsic motivation score, with higher scores indicating stronger intrinsic regulation of exercise behaviour. Previous studies have demonstrated good psychometric properties of the Lithuanian version of instrument (Baceviciene & Jankauskiene, 2021). The internal consistency at pre-test, post-test and follow-up was good (Cronbach’s α = 0.90; 0.88 and 0.93 respectively).

### 2.4. Statistical Analysis

All statistical analyses were conducted using IBM SPSS Statistics (v.31, IBM Corp., Armonk, NY, USA). The level of significance was set at *p* < 0.05. Descriptive statistics (means ± SD) were computed for all variables. The normality of each variable was verified using the Shapiro–Wilk test, as well as through inspection of histograms and Q–Q plots. All variables demonstrated approximately normal distributions, as indicated by non-significant Shapiro–Wilk test results for BAS-2 (*p* = 0.601), FAS (*p* = 0.225), Body and Mind subscale of the PABEQ (*p* = 0.457), Body Surveillance from OBC (*p* = 0.764), intrinsic exercise regulation (*p* = 0.388), and leisure-time exercise score (*p* = 0.153), supporting the use of parametric statistical analyses.

Internal consistency of multi-item scales was evaluated using Cronbach’s α. Alpha values ≥ 0.70 were interpreted as acceptable, values ≥ 0.80 as good, and values ≥ 0.90 as excellent (Cronbach, 1951; Vaske et al., 2017). 

One-way ANOVA was performed to assess potential baseline differences among the three groups, while an independent sample *t*-test was used to compare study measures between completers and non-completers after the follow-up.

A priori power analysis was conducted at the study design stage to ensure adequate power to detect the primary intervention effect, operationalized as the group × time interaction in a 3 (group: functional training, Nirvana Fitness, control) × 3 (time: baseline, 8-week post-intervention, 12-month follow-up) longitudinal design. The required sample size was estimated using G*Power (v.3.1.9.7, Windows) (F tests: repeated-measures ANOVA, within–between interaction), assuming α = 0.05, power (1 − β) = 0.80, and a medium interaction effect (Cohen’s *f* = 0.25), with a correlation among repeated measures of r = 0.50. This calculation indicated a minimum total sample of approximately ≈ 45 participants. The final sample comprised 48 participants (functional training n = 15; Nirvana Fitness n = 16; control n = 17). Although the power calculation was based on the repeated-measures ANOVA framework for planning purposes, analyses were conducted using linear mixed-effects models, which test the same group × time effect while appropriately accounting for within-participant correlation and missing data.

Changes in outcome variables over time and differences between intervention groups were examined using linear mixed-effects models. Separate models were fitted for each outcome variable (body appreciation, functionality appreciation, body–mind connection, body surveillance, intrinsic exercise motivation, and leisure-time exercise). Group (functional training, Nirvana Fitness, control), time (baseline, 8-week post-intervention, 12-month follow-up), and their group × time interaction was included as fixed effects. Age and body mass index (BMI) were entered as covariates in all models.

To account for the longitudinal structure of the data, participants were treated as random effects by including a random intercept for each participant. Repeated measurements within individuals were modelled using an unstructured covariance matrix. Models were estimated using restricted maximum likelihood (REML). Fixed effects were evaluated using Type III F tests, with degrees of freedom estimated using the Satterthwaite approximation. When significant group × time interactions were observed, interpretation focused on these interaction effects, supported by estimated marginal means and their 95% confidence intervals. Estimated marginal means adjusted for covariates were used for the graphical presentation of trajectories over time. Statistical significance was set at *p* < 0.05.

## 3. Results

Table 1 presents the baseline comparison of the study variables across the Functional Training (FT), Nirvana Fitness (NF), and Control (CN) groups. One-way ANOVA results indicated that, at baseline, there were no significant group differences in body appreciation, body surveillance, leisure-time exercise, or perceived physical fitness. However, significant between-group differences were observed for functionality appreciation, body–mind connection, and intrinsic exercise motivation. Specifically, participants in the NF and control groups demonstrated significantly higher functionality appreciation, body–mind connection, and intrinsic motivation scores than those in the FT group (*p* < 0.05, Sidak-adjusted post hoc tests).

Table 2 summarises unadjusted means and standard deviations at baseline, post-intervention, and 12-month follow-up for each group. These descriptive data provide an overview of the sample characteristics and observed score distributions and serve as context for the linear mixed-effects model analyses reported below.

Results of the linear mixed-effects models are presented in Table 3. For body appreciation (BAS-2), a significant main effect of time and a significant group × time interaction was observed. Inspection of the estimated marginal means indicated that body appreciation increased from baseline to post-intervention in both intervention groups, with these higher levels largely maintained at the 12-month follow-up, whereas the control group showed relatively stable values across time. For functionality appreciation (FAS), significant main effects of time and a significant group × time interaction were found. Estimated marginal means suggested increases following the intervention in the functional training and Nirvana Fitness groups, with smaller changes observed in the control group. For the body–mind connection, significant main effects of group and time were detected, along with a significant group × time interaction. The interaction reflected greater improvements over time in the intervention groups compared with the control group. For body surveillance, a significant main effect of time was observed, while group and group × time effects were not significant, indicating that changes from baseline through post-intervention and follow-up occurred similarly across groups. For leisure-time exercise, a significant main effect of group was observed, indicating overall differences in activity levels between groups when averaged across time points. However, neither the main effect of time nor the group × time interaction was significant, suggesting no evidence of change over time or differential change between groups. For intrinsic exercise regulation, significant main effects of time and a significant group × time interaction were found. Examination of marginal means showed increases in intrinsic regulation from baseline to post-intervention in the intervention groups, whereas changes in the control group were minimal.

**Figure 2:**
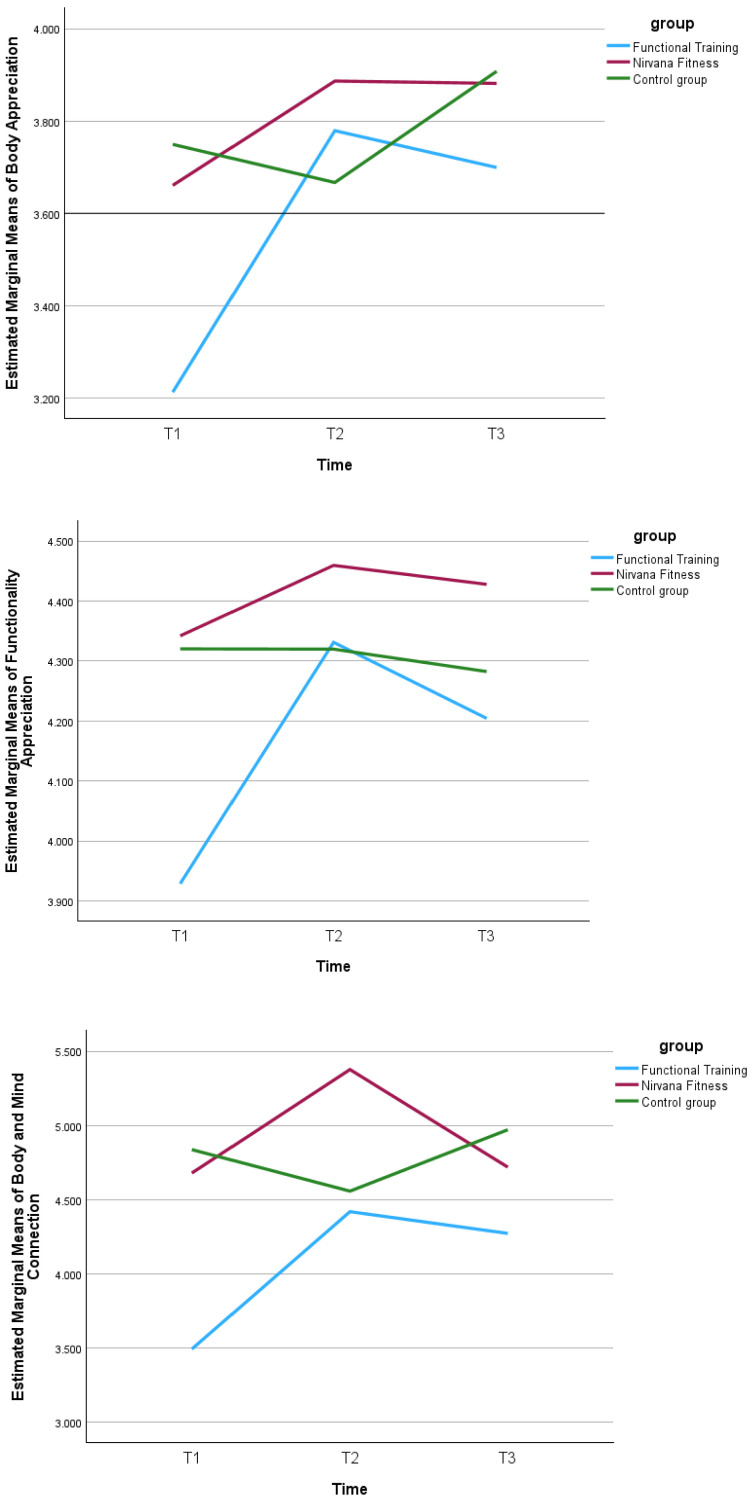

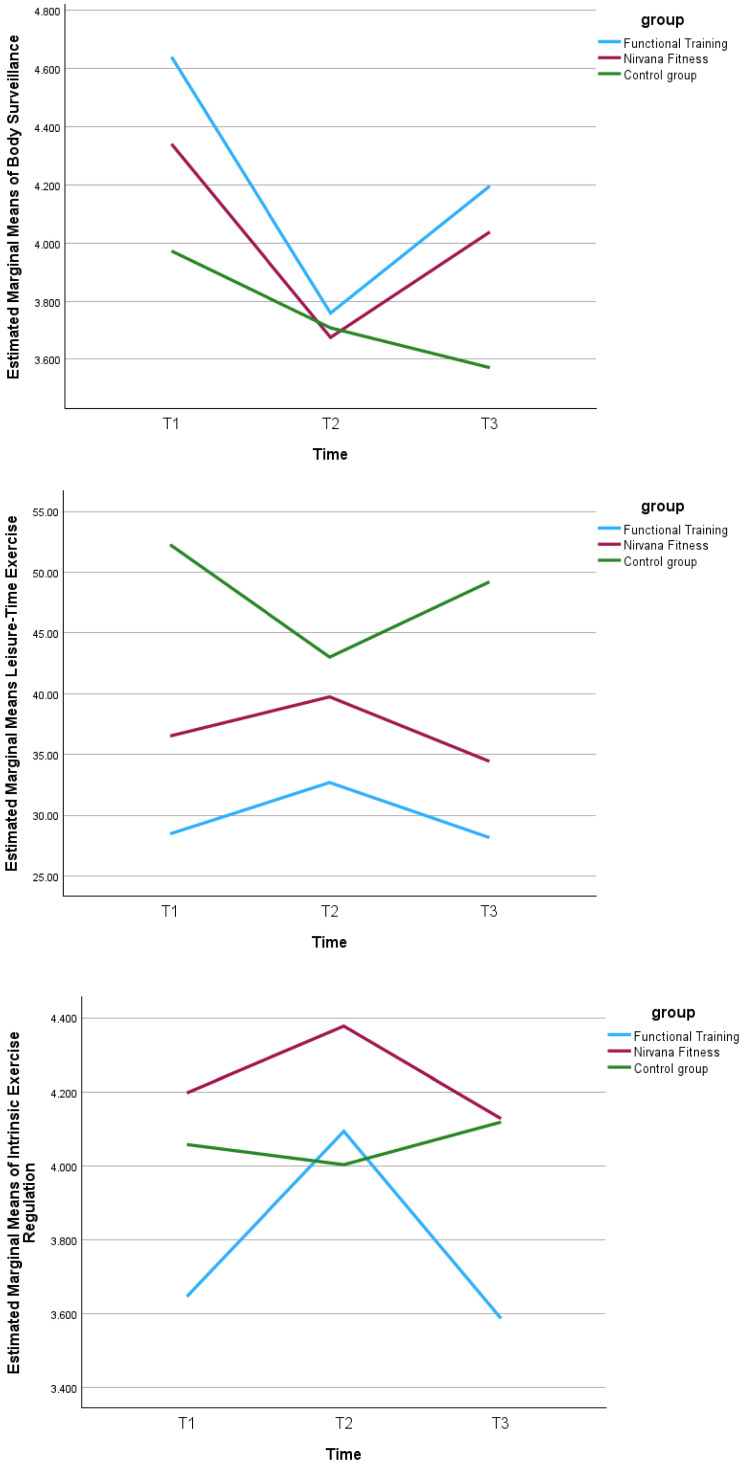
Group × time interaction in estimated marginal means of body appreciation, functionality appreciation, body–mind connection, body surveillance, leisure-time exercise, and intrinsic exercise regulation. Note: estimated marginal means derived from linear mixed-effects models. T1 = baseline assessment; T2 = 8-week post-intervention assessment; T3 = 12-month follow-up. Estimates are adjusted for age and body mass index.

Group × time interaction in estimated marginal means of study variables are presented in Figure 2. 

## 4. Discussion

This study builds on emerging research on exercise-based approaches to positive body image, extending existing knowledge by examining the sustainability of psychological and motivational outcomes one year after two contemporary movement-based programmes—Functional Training and Nirvana Fitness—in young women. A significant contribution of this work is the demonstration that enhancements in positive body image-related constructs can endure over an extended period following a relatively brief intervention, even in the absence of ongoing support. Concurrently, the findings underscore that enhancements in positive body image do not invariably translate into enduring modifications in physical activity behaviour. 

Specifically, the results indicated greater and more sustained improvements in body appreciation, functionality appreciation, and body–mind connection in the intervention groups compared with the control group. Few intervention studies beyond yoga-based programmes have included follow-up assessments that allow for direct comparison with the present findings. Nevertheless, the results are consistent with previous evidence showing that mindfulness-based physical activity can positively influence body appreciation and body–mind connection (Alleva et al., 2020; Halliwell et al., 2019). With respect to the sustainability of change, one prior study reported that a brief, four-session yoga programme enhanced body appreciation and body connection, with effects persisting at a four-week follow-up (Halliwell et al., 2019). 

Potential mechanisms underlying these changes may be understood within the Developmental Theory of Embodiment (Piran & Neumark-Sztainer, 2021) which posits that body connection, experiences of functionality and agency, pleasure and vitality, relative freedom from self-objectification, and supportive relational contexts are central components of positive embodiment. The present findings suggest that physical activity contexts that support these principles may facilitate sustained improvements in positive body image.

In addition, the observed sustainability of changes may also be interpreted through the lens of the Monitoring and Acceptance Theory (Lindsay & Creswell, 2017) which proposes that mindfulness operates through two complementary processes: attentional monitoring and non-judgemental acceptance of internal experiences. Professionally delivered physical activity emphasising breath regulation and mindful movement is likely to engage these processes by directing attention toward present-moment bodily sensations (Cook-Cottone et al., 2020; Ullrich-French et al., 2022). Such engagement may enhance interoceptive awareness and positive embodiment (e.g., stronger body–mind connection) and reduce appearance-focused evaluative thoughts (e.g., body surveillance). Increase in body and mind connection and interoceptive awareness might also explain sustainable changes in positive body image after participation in FT (Menzel & Levine, 2011; Wallman-Jones et al., 2021). However, in the present study we did not measure interoceptive awareness and future studies are recommended to do so. 

In the present study, improvements in body surveillance were not maintained at the follow-up assessment. This attenuation may partly reflect limited statistical power or regression to the mean, particularly given the relatively small sample size and repeated-measures design. Importantly, the absence of robust group differences at follow-up should not be interpreted as definitive evidence of a lack of sustainability, but rather as an indication that sustainable changes in these outcomes may be more difficult to detect under the current study conditions.

Positive body image has been linked to intrinsic exercise regulation and health-related exercise goals (Hurst et al., 2017; Tylka & Wood-Barcalow, 2015b). A systematic review further concluded that higher-quality, autonomous forms of exercise motivation are associated with more favourable body image outcomes (Panão & Carraça, 2020). In the present study, levels of intrinsic exercise motivation were maintained over the 12-month follow-up period. This finding is noteworthy given that intrinsic motivation is associated with sustained engagement in exercise and higher levels of physical activity (Ryan et al., 2009). Taken together, these results provide preliminary evidence that mindful, body functionality oriented and body image–safe exercise interventions may help support the sustainable increase and maintenance of post-intervention levels of intrinsic exercise motivation. However, further research is needed to confirm these findings. 

Despite significant psychological benefits being observed in both intervention groups, neither programme produced any changes in leisure-time physical activity, either in the short or long term. Previous research suggests that participation in structured exercise does not necessarily translate into higher overall physical activity, as individuals may adjust their activity levels outside the intervention sessions (Wasenius et al., 2014). However, such compensatory effects were not directly assessed in the present study. Additionally, although FT and NF were designed to enhance relaxation and body awareness through functionality and mindfulness, they did not incorporate behavioural change techniques (e.g., goal setting, social support, or environmental restructuring) that are often considered important for sustaining physical activity behaviour (Hagger, 2019; Madigan et al., 2024; Strobach et al., 2020). Sustained increases in physical activity may also require ongoing reinforcement or booster sessions (Tarantino et al., 2025) which were not included in the current intervention. Finally, external constraints (e.g., time pressures and academic demands) and limitations of self-reported measures such as the LTEQ may have contributed to the observed null findings. 

### 4.1. Practical Implications and Future Directions

The findings of this study suggest several potential practical implications for exercise professionals, health educators, and programme developers interested in supporting positive body image among young women. Both FT and NF were associated with sustained improvements in constructs related to positive body image. These results indicate that body image–safe, structured exercise programmes that emphasise body functionality and mindfulness may contribute to sustainable improvements in young women’s positive body image. However, the absence of observed changes in leisure-time physical activity highlights the potential need for strategies that extend beyond the initial intervention period to support behavioural change. Such approaches might include ongoing participation opportunities, periodic booster sessions, or low-intensity digital follow-up components aimed at supporting continued engagement in exercise. Previous reviews suggest that booster contacts (e.g., phone calls or text messages) may help support the maintenance of physical activity over time (Tarantino et al., 2025). Given the bidirectional relationship between body image and physical activity (Sabiston et al., 2019), such strategies may also be valuable in maintaining positive body image improvements over time. 

### 4.2. Limitations and Strengths of the Study

Several important limitations should be considered when interpreting the findings of this study. First, the relatively small sample size may have limited statistical power to detect changes in some outcomes at the 12-month follow-up. Future research with larger samples and extended follow-up periods is therefore warranted. Second, no active follow-up engagement or booster sessions were provided during the 12-month period due to resource constraints. While this absence allowed us to observe outcome trajectories under naturalistic conditions, it may also have contributed to the partial attenuation of some effects over time. Third, the quasi-experimental, non-randomised design restricts causal inference regarding the effects of the Functional Training and Nirvana Fitness interventions. Participants self-selected into the study and were allocated to groups based on practical considerations rather than randomisation, which may have introduced self-selection and allocation bias. As a result, baseline differences in motivation, interest in exercise, or body image–related characteristics cannot be ruled out as contributors to the observed outcomes. Fourth, neither participants nor instructors were blinded to group allocation, which may have increased the risk of expectancy or social desirability effects, particularly for self-reported psychological outcomes. In addition, all outcome measures relied on self-report, which may be subject to recall bias. Finally, missing data at follow-up were present, as is common in longitudinal research. Although appropriate statistical approaches were used to maximise data retention, attrition may still have influenced the findings if dropout was non-random. Moreover, adherence to the structured exercise participation during the follow-up period were not systematically assessed, and engagement in other exercise activities may have affected the outcomes. Future studies should monitor physical activity behaviour, intervention exposure, and reasons for attrition throughout follow-up.

Despite these limitations, the study has several strengths that enhance the credibility of its findings and its contribution to the field. Few studies in positive body image research have examined whether exercise-based interventions produce sustained benefits for women and girls beyond the immediate post-intervention period (Halliwell et al., 2019; Matheson et al., 2023) and under naturalistic conditions. By following participants for 12 months after the end of the intervention, the present study provides rare evidence regarding the sustainability of positive body image changes. In addition, although the primary aim was to examine the maintenance of positive body image–related changes, the study also assessed the sustainability of leisure-time physical activity, an outcome that is infrequently evaluated in this area of body image research (Matheson et al., 2023). 

## 5. Conclusions

This quasi-experimental study provides exploratory evidence regarding the sustainability of positive body image–related outcomes one year after an 8-week body functionality– and mindfulness-oriented movement interventions in young women. Participation in the NF and FT programmes was associated with sustained improvements in body appreciation, functionality appreciation, body–mind connection, and intrinsic exercise motivation. In contrast, changes in body surveillance were not maintained over time, and neither programme was associated with increases in leisure-time physical activity behaviour compared with the control group. These findings provide initial evidence that participation in professionally delivered, structured, body image–safe exercise programmes that prioritise body functionality and mindfulness may be related to sustained improvements in positive body image among young women. Nevertheless, these findings should be interpreted with caution given the exploratory nature of the study, the quasi-experimental design, and the relatively small sample size, which limit generalisability. Future research employing randomised controlled designs and larger, samples is needed to confirm and extend these findings.

## Figures and Tables

**Table 1:** The comparison of the study measures between groups at the baseline (means ± SD).

Variables	Functional Training (n = 15)	Nirvana Fitness (n = 16)	Control Group (n = 17)	F	ŋ^2^	*p*
BAS-2	3.32 ± 0.78	3.75 ± 0.78	3.61 ± 0.80	1.19	0.05	0.315
FAS	3.85 ± 0.41	4.43 ± 0.61 *	4.32 ± 0.55 *	5.19	0.19	0.009
Body–Mind ^a^	3.23 ± 0.97	4.76 ± 1.24 *	4.86 ± 1.44 *	8.43	0.27	<0.001
BS	4.47 ± 1.41	4.33 ± 0.81	4.15 ± 0.89	0.37	0.02	0.694
LTEQ	24.33 ± 18.60	39.81 ± 28.79	50.82 ± 46.37	2.44	0.10	0.098
IEM	3.23 ± 0.90	4.39 ± 0.75 *	3.99 ± 0.75 *	8.32	0.27	<0.001

Note. SD—standard deviation, BAS-2—Body Appreciation Scale 2, FAS—Functionality Appreciation Scale, ^a^ Body and Mind Connection Subscale from the Physical Activity Body Experiences Questionnaire, BS—Body Surveillance Subscale from the Objectified Body Consciousness Scale, LTEQ—Leisure-Time exercise Questionnaire score, IEM—Intrinsic Exercise Regulation from the Behavioral Regulation in Exercise Questionnaire 2; F—Fisher statistics from the One-Way ANOVA, ŋ^2^—eta squared effect size; * *p* < 0.05 as compared to the Functional Training group (post hoc Sidak test).

**Table 2:** Descriptive statistics (mean ± SD) of outcome variables by group and time.

Measures	Functional Training (n = 15)			Nirvana Fitness (n = 16)			Control Group (n = 17)		
	T1	T2	T3	T1	T2	T3	T1	T2	T3
BAS-2	3.32 ± 0.78	3.86 ± 0.52	3.77 ± 0.75	3.75 ± 0.78	3.99 ± 0.64	3.96 ± 0.77	3.61 ± 0.80	3.37 ± 0.89	3.70 ± 0.76
FAS	3.85 ± 0.41	4.36 ± 0.49	4.19 ± 0.54	4.43 ± 0.61	4.63 ± 0.45	4.54 ± 0.53	4.32 ± 0.55	4.33 ± 0.56	4.29 ± 0.64
Body–Mind ^a^	3.23 ± 0.97	4.20 ± 1.18	4.09 ± 1.76	4.76 ± 1.24	5.48 ± 0.92	4.79 ± 1.27	4.86 ± 1.44	4.39 ± 1.03	4.91 ± 0.98
BS	4.47 ± 1.41	3.68 ± 0.92	4.08 ± 1.28	4.33 ± 0.81	3.73 ± 0.96	4.06 ± 0.90	4.15 ± 0.89	4.08 ± 1.03	3.79 ± 1.18
LTEQ	24.33 ± 18.60	29.67 ± 17.94	25.53 ± 23.99	39.81 ± 28.79	43.50 ± 25.83	37.19 ± 29.58	50.82 ± 46.37	39.82 ± 23.77	47.24 ± 33.69
IEM	3.23 ± 0.90	3.87 ± 1.19	3.28 ± 1.31	4.39 ± 0.75	4.64 ± 0.63	4.36 ± 0.82	3.99 ± 0.75	4.03 ± 0.72	4.10 ± 0.85

Note: values are unadjusted means ± standard deviations (SD). T1 = baseline, T2 = 8-week post-intervention, T3 = 12-month follow-up. BAS-2—Body Appreciation Scale 2, FAS—Functionality Appreciation Scale, ^a^ Body and Mind Connection Subscale from the Physical Activity Body Experiences Questionnaire, BS—Body Surveillance Subscale from the Objectified Body Consciousness Scale, LTEQ—Leisure-Time Exercise Questionnaire score, IEM—Intrinsic Exercise Regulation from the Behavioral Regulation in Exercise Questionnaire 2.

**Table 3:** Linear mixed-effects model results for intervention effects.

Outcome	Group F (df)	*p*	Time F (df)	*p*	Group × Time F (df)	*p*
BAS-2	0.77 (2, 112.00)	0.467	10.15 (2, 61.68)	<0.001	6.34 (4, 61.54)	<0.001
FAS	1.66 (2, 98.65)	0.196	5.26 (2, 61.35)	0.008	2.75 (4, 61.32)	0.036
Body–Mind ^a^	3.97 (2, 108.60)	0.022	9.59 (2, 61.94)	<0.001	9.16 (4, 61.87)	<0.001
BS	1.91 (2, 107.77)	0.153	18.13 (2, 61.16)	<0.001	2.23 (4, 61.14)	0.076
LTEQ	6.00 (2, 103.76)	0.003	0.10 (2, 65.44)	0.909	1.15 (4, 65.32)	0.340
IEM	1.88 (2, 108.24)	0.158	4.18 (2, 60.19)	0.020	2.94 (4, 60.22)	0.027

Note: Age and body mass index are entered as covariates. BAS-2—Body Appreciation Scale 2, FAS—Functionality Appreciation Scale, ^a^ Body and Mind Connection Subscale from the Physical Activity Body Experiences Questionnaire, BS—Body Surveillance Subscale from the Objectified Body Consciousness Scale, LTEQ—Leisure-Time Exercise Questionnaire score, IEM—Intrinsic Exercise Regulation from the Behavioral Regulation in Exercise Questionnaire 2; F—F statistics from linear mixed-effects models, df—numerator and denominator degrees of freedom.

## Data Availability

The original contributions presented in this study are included in this article. Further inquiries can be directed to the corresponding author.
